# Autonomic Neuropathy in Diabetes Mellitus

**DOI:** 10.3389/fendo.2014.00205

**Published:** 2014-12-01

**Authors:** Alberto Verrotti, Giovanni Prezioso, Raffaella Scattoni, Francesco Chiarelli

**Affiliations:** ^1^Department of Pediatrics, University of Perugia, Perugia, Italy; ^2^Department of Pediatrics, University of Chieti, Chieti, Italy

**Keywords:** diabetic autonomic neuropathy, diabetes mellitus, autonomic nervous system, hyperglycemia, oxidative stress, advanced glycation end-products, inflammation, cardiovascular autonomic neuropathy

## Abstract

Diabetic autonomic neuropathy (DAN) is a serious and common complication of diabetes, often overlooked and misdiagnosed. It is a systemic-wide disorder that may be asymptomatic in the early stages. The most studied and clinically important form of DAN is cardiovascular autonomic neuropathy defined as the impairment of autonomic control of the cardiovascular system in patients with diabetes after exclusion of other causes. The reported prevalence of DAN varies widely depending on inconsistent definition, different diagnostic method, different patient cohorts studied. The pathogenesis is still unclear and probably multifactorial. Once DAN becomes clinically evident, no form of therapy has been identified, which can effectively stop or reverse it. Prevention strategies are based on strict glycemic control with intensive insulin treatment, multifactorial intervention, and lifestyle modification including control of hypertension, dyslipidemia, stop smoking, weight loss, and adequate physical exercise. The present review summarizes the latest knowledge regarding clinical presentation, epidemiology, pathogenesis, and management of DAN, with some mention to childhood and adolescent population.

## Definition

Diabetic neuropathy is the main cause of neuropathy in the world (1). As one of the major complications (2), it plays a key role in morbidity and mortality in patients with type 1 and type 2 diabetes mellitus (T1DM and T2DM).

Diabetic neuropathy is classically defined as “the presence of symptoms and/or signs of peripheral nerve dysfunction in people with diabetes after the exclusion of other causes” (3). Sensory, motor, or autonomic nerves can be involved, often coexisting.

The Thomas and Boulton classifications distinguish between generalized symmetric polyneuropathies (DPNs) and focal/multifocal neuropathies (4, 5).

Diabetic autonomic neuropathy (DAN) is included in the first group. Erroneously considered for a long time in the past century as a rare event, DAN is indeed a serious and often underestimated complication of diabetes for two main reasons: by potentially affecting any circuit/tract of autonomic nervous system, DAN is a systemic-wide disorder, which encompasses a large spectrum of organs and leads to significant increase in morbidity and mortality (6–8); moreover DAN in early stages may be asymptomatic, especially in young T1DM patients, often compromising early diagnosis and treatment.

In fact, subclinical DAN can occur within a year of diagnosis in T2DM and within 2 years in T1DM, while first symptoms may onset after years (6, 9, 10).

### Cardiovascular autonomic neuropathy

The most common and studied manifestation of DAN is cardiovascular autonomic neuropathy (CAN), owing to its life-threatening complications (arrhythmias, silent myocardial ischemia, and sudden death) and to its relation with other microangiopathic comorbidities. CAN is defined as the impairment of autonomic control of the cardiovascular system (5). In recent years, much attention has been directed to early warning signs of CAN, detectable in the first years after diabetes onset by means of validated cardiovascular reflex tests (11) supported by newer procedures (12–14). Such warning signs include reduced heart rate (HR) variability during deep breath, prolongation of QT interval, temporally followed by resting tachycardia, impaired exercise tolerance, and decreased baroreflex sensitivity with consequent abnormal blood pressure regulation, and orthostatic hypotension (12, 15).

A recent cross-sectional study on 387 diabetic adult patients showed that there was a tendency toward increased CAN prevalence with increased resting HR and highlighted the importance of resting HR as a predictive value for CAN (16). Despite the evidence of the increase in CAN severity with diabetes duration, a study on 684 T1DM adult patients has recently reported that diabetes duration by itself was not a good predictor of CAN severity (17).

Cardiac alterations initially start with a relative increase of the sympathetic tone, since diabetic neuropathy firstly affects longest fibers as those of parasympathetic system (like the vagus nerve). Sympathetic denervation begins at the following stage, by affecting the heart from the apex toward the base, gradually impairing ventricle function and resulting in cardiomyopathy (12).

### Other manifestations of DAN

The central control of breathing and the sympathetic bronchial innervation can also be jeopardized by the autonomic impairment. Both peripheral and central chemosensitivity to hypoxia is altered, as is the bronchomotor tone in lung. The coexistence of this finding with other risk factors like lung microvascular complications, endocrine impairments, obesity, and hypertension, lead to a higher prevalence of sleep apnea syndrome (SAS) in diabetic patient (18, 19). Clinical implications of SAS go from a decrease in quality of life due to sleepiness to an increased risk of sudden death (20). The meta-analysis of Fujihara et al. indicated that patients with DAN had about twofold higher prevalence of SAS than patients without DAN, the association being more remarkable among young patients with T2DM (21).

Furthermore, DAN may manifest with gastrointestinal (GI) symptoms, as a result of the remodeling of the enteric nervous system (ENS) induced by diabetes (22). Loss in inhibitory and increase in excitatory enteric neurons, as well as decrease in sensory neuropeptides, may induce gastroparesis, esophageal dysmotility, constipation, diarrhea, fecal incontinence, or gallbladder atony. In general, the presence of gastroparesis weakly correlates with upper GI autonomic symptoms (nausea, vomiting, early satiety, postprandial fullness, bloating, and abdominal pain), which are very common in T1DM and T2DM patients. Nevertheless, it has been reported (23) that gastric dysmotility has an impact in acute glycemic control by delaying glucose absorption while, on the other hand, acute glycemic imbalance may lead to temporary functional GI abnormalities.

The earlier damages to the sacral parasympathetic fibers contributes to genitourinary dysfunction, starting from impaired bladder sensation with increase in urine retention to dysuria, nicturia, incomplete bladder emptying, and urgency up to overflow incontinence due to the progressive involvement of motor sympathetic and somatic nerves (5, 7). Bladder dysfunction as well predisposes to recurrent urinary tract infections and may be predictive for long-term development of renal failure.

Diabetic autonomic neuropathy along with vasculopathy, connective tissue damage, and other psychological, endocrine, nutritional, and pharmacological factors may influence sexuality, by inducing erectile dysfunction, retrograde ejaculation and decreased sexual desire in female, dyspareunia, or inadequate lubrication (24–26).

Autonomic pupillomotor function and sudomotor function are not spared by DAN. The sympathetic predominance in pupil control decreases its diameter at rest (27). Preserved pupil miotic reaction to accommodation-convergence without the miotic reaction to light is named “Argyll-Robertson pupil,” a clinical sign shared with neurosyphilis. Sweat gland denervation results in skin dryness, which is strongly linked to the development of typical foot ulcerations (28).

## Epidemiology

The reported prevalence of DAN varies widely depending on different criteria used to define autonomic dysfunction, different type and number of tests performed, the use of age-related normative values, the presence or absence of signs and symptoms of autonomic neuropathy, and different patient cohorts studied (5, 14, 29–33).

A meta-analysis of adult patients including 15 studies from 1966 to 2001 reported prevalence rates of CAN between 1 and 90% (30, 34), while Dimitropoulos reported a prevalence of CAN that varies between 1 and 90% in patients with T1DM and 20–70% in patients with T2DM (14).

On the other hand, in a community-based population study, the prevalence of autonomic neuropathy, defined by one or more abnormal HR variability test results was 16.7% (34, 35).

In 1992, Ziegler et al. in a multicenter study reported that the prevalence of CAN in T1DM and T2DM patients was 25.3 and 34.3%, respectively (more than two of six abnormal autonomic function tests). Using more strict criteria (abnormalities in at least three of six autonomic function tests), the prevalence of CAN was 16.8% for patients with type 1 and 22.1% for individual with T2DM (30, 36) and a similar prevalence rate had been found by O’Brien et al. in patients with T1DM (30, 37).

In our experience (38), 47 of 110 diabetic children and adolescents showed one or more abnormal test for cardiovascular autonomic dysfunction, while in a prospective study, Solders et al. reported low sensory nerve conduction and autonomic dysfunction in about 25% of 144 diabetic children (39), instead Karavanaki found evidence of reduced papillary adaptation in darkness in 13.8% of children with diabetes compared with 5.8% of controls, 50% of these children also had impaired HR variation (40).

The Diabetes Control and Complications Trial (DCCT) found that 1.65% had abnormal HR variability at baseline in patients with <5 years duration of diabetes. The prevalence increased to 6.2% in patients with more than 5 but <9 years duration of diabetes and to 12.2% by 9 years duration of diabetes (30, 41).

The prevalence of confirmed CAN (defined as the abnormality of at least two cardiovascular HR results) in clinical studies in unselected populations, including both T1DM and T2DM patients, varies from 16.6 to 20% (31, 42, 43), and this prevalence may increase to 65% with increasing age and diabetes duration (29, 31). In particular, the prevalence may increase up to 38% in T1DM and 44% in T2DM patients aged 40–70 years and up to 35% in T1DM and 65% in T2DM patients with long standing diabetes (31, 33, 44, 45).

Cardiovascular autonomic neuropathy is detected in about 7% of both T1DM and T2DM at the time of diagnosis. The annual increase in prevalence of CAN has been reported about 6% in T2DM and 2% in T1DM (14, 31, 33, 45, 46).

Diabetic autonomic neuropathy may also cause GI disturbances, affecting every part of the GI tract: delayed esophageal transit (50%), gastroparesis (40%), disordered small and large intestinal motility with diarrhea (20%) and constipation (25%) (5, 30).

The prevalence of organic sexual dysfunction is also high with erectile dysfunction (35–90%) and retrograde ejaculation (32%) (5, 30).

Bladder dysfunction is detected in 43–85% of patients with T1DM and in 25% of T2DM (5, 30).

Discussing about pediatric population, a systematic review by Tang including 19 studies about young people with T1DM, reported a prevalence of DAN that varied between 16 and 75% for cardiovascular nerve function tests and between 8 and 16% in pupillometry studies (27). Furthermore, several studies reported a significant association between glycemic control, longer diabetes duration, and autonomic test abnormalities in young people (27). Only few studies analyze the association between DAN and other microvascular complications in young people with diabetes: retinopathy and nephropathy seems to be associated with DAN data from adult population (27).

## Pathogenesis

### Hyperglycemia and oxidative stress

**Figure 1:**
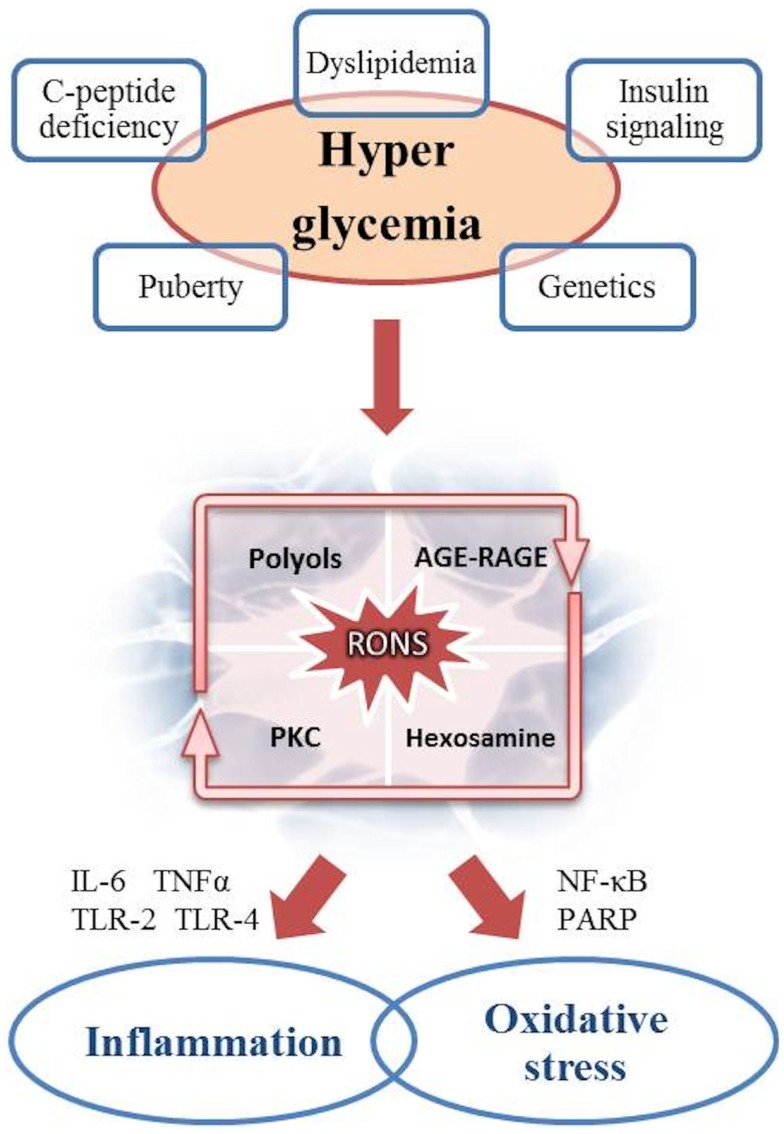
**Pathogenesis of DAN: the role of hyperglycemia as the cause of inflammation and oxidative stress**.

An increasing body of data supports the multifactorial genesis of DAN (Figure 1).

It is well established that hyperglycemia is the main driver of diabetic complications. The increase in blood and cytoplasmic glucose induces several metabolic pathways into a vicious cycle resulting in chronic tissue damage. Within the cell, the mitochondrial overproduction of reactive oxygen and nitrogen species (RONS) like superoxide anion radical, peroxynitrite, and hydrogen peroxide is the key event secondary to glucose overload (47). Longitudinal studies showed an higher prevalence of oxidative stress in female and an increased risk for sensory dysfunction, CAN, and mortality (48, 49). RONS in turn, induce DNA damage and consequently overstimulate the poly-ADP ribose polymerase (PARP), a repair enzyme inducing NAD consumption and decreasing glyceraldehyde 3-phosphatedehydrogenase (GAPDH) activity, already impaired by RONS. This results in endothelial dysregulation and pro-apoptotic signals initiation, like advanced glycation end products (AGEs) formations. The production of AGEs leads to structural and functional protein alteration both in the extracellular matrix and in the intracellular space. AGEs may also interact with specific receptors (RAGEs), which create a complex pro-inflammatory cascade (involving IL-1, IL-6, TNF-α, TGF-β, and VCAM-1) and increase oxidative stress (14, 47, 48, 50, 51). It is noteworthy that experimental studies on RAGE knockout mice have demonstrated a reduced frequency in neuronal complications (52).

Protein kinase C (PKC) and hexosamine pathways are thus enhanced by the impaired cellular milieu, respectively, resulting in further production of RONS by means of NADPH oxidase complex, and in transcription of vascular impairment factors, like PAI-1, TGF-α, and TGF-β (50).

Furthermore, glucose in excess is converted into polyols, particularly sorbitol, whose increased flux consumes NADPH, involved in antioxidant regeneration. Polyols accumulation also inhibits Na^+^/K^+^ ATPase, thus interacting with PKC pathway.

### Inflammation

The role of inflammation in the pathogenesis of DAN has increasingly been highlighted. Adhesion molecules expression, cytokine overproduction, phagocytic cells infiltration, and innate immune system activation via toll-like receptors (TLR-2 and TLR-4) cause secondary neuronal and vascular damage, also by determining a continuous cross-talk with the oxidative stress (53, 54). An increase in traditional circulating inflammatory markers like C-reactive protein (CRP), IL-6, IL-8, TNF-α, and endothelin-1, as well as novel markers like the urokinase plasminogen activator receptor (SuPAR), have repeatedly been detected in several studies on T1DM and T2DM (55–61). These markers have been considered predictive for diabetic complications, especially CAN (62).

### Other factors

With reference to T1DM, the role of autoimmunity has also been considered. Autoantibodies against sympathetic ganglia, vagus nerve, and adrenal medulla were found in T1D patients (63, 64). Recent studies have shown that these antibodies were independent from islet autoimmunity while the data about the predictivity for future development of DAN and CAN are conflicting (65, 66).

Microvascular damage itself should not be underestimated as an indirect factor of neuronal dysfunction and apoptosis (14, 67). Even an impaired dynamic cerebral autoregulation has been linked to CAN (68).

Over the past decade, new pathogenic theories closely interrelated to the classic mechanisms have been suggested. A genetic predisposition to oxidative stress and an increased risk in neuropathy due to polymorphism of antioxidant enzymes like superoxide dismutase (SOD), glutathione peroxidase (GPX), and catalase (CAT) has been demonstrated in a diabetic population (69).

Great interest has been generated by the role of nerve growth factors in the pathogenesis of DAN: for instance, insulin-like growth factor-1 (IGF-1) and neurotrophin-3 (NT-3) have been demonstrated to reverse experimental diabetic neuropathy (70).

Besides the hyperglycemia, of particular interest are the studies on dyslipidemia showing the free fatty acids being key mediators of inflammation and oxidative damage, as well as elevation in plasma triglycerides or decrease in HDL cholesterol correlating to diabetic neuropathy (67, 71). Furthermore, in experimental studies, leptin receptor deletion in mice resulted in autonomic neuropathy (72).

Puberty can also have a certain pathogenetic importance (14, 73, 74): it appears to decrease insulin sensitivity, especially by rising GH and reducing IGF-1 circulating levels. This may alter the metabolic profile to promote microvascular complications (32, 75, 76). Moreover, a poor compliance to therapy and diet should not be underestimated during adolescence in consequence of neuropsychological problems.

## Prevention

Intensive glycemic control seems to be the most effective way to prevent/delay the onset and slow the progression of autonomic dysfunction in patients with T1DM (14, 30, 33, 46, 77). The DCCT demonstrated that intensive glycemic treatment reduces the onset and the progression of diabetic complications (retinopathy, nephropathy, and neuropathy) and also reduces the rate of CAN by 53% compared to conventional therapy (41, 45, 78 79). The Epidemiology of Diabetes Intervention and Complication (EDIC) study has demonstrated persistent beneficial effects of early strict glycemic control on microvascular complications and also on DAN: although CAN prevalence increased in both groups, the former intensive insulin treatment group continued to have a significantly lower decline in CAN through 13 or 14 years of EDIC follow-up. These long-term beneficial effects of early intensive glucose control have been called “metabolic memory” (14, 30, 33, 45, 80–83). A 3 year prospective trial, by the use of PET cardiac imaging, has shown a similar beneficial effect of good glycemic control (defined by HbA1c <8%) on autonomic function (84).

The EURODIAB IDDM complication study and the EURODIAB Prospective study indicated that, apart from glycemic control, the incidence of DAN in patients with T2DM may be associated with potentially modifiable cardiovascular risk factors including a raised triglyceride level, body-mass index, smoking, and hypertension. This finding may be important for the development of risk reduction strategies (67, 85).

In T2DM, intensive glycemic control seems to be less effective in prevention of autonomic function deterioration (14, 33). During the VA Cooperative Study, no difference in the prevalence of autonomic neuropathy derived by the use of intensive insulin therapy and strict glycemic control in patients with T2DM (86). Three large studies (VADT, ACCOR, and ADVANCE) have reported that intensive glycemic control does not reduce all-cause mortality while increases hypoglycemic episode frequency (87–89).

The best results on prevention of autonomic dysfunction in patients with T2DM seem to be derived by multifactorial strategy treatment and lifestyle modification. Steno-2 study showed that a multifactorial cardiovascular risk intervention based on behavioral therapy (diet, smoking cessation, and physical exercise) and pharmacological intervention (to control hypertension, dyslipidemia, and hyperglicemia) retards development and progression of CAN in patients with T2DM and microalbuminuria (90). In the Diabetes Prevention Program (DPP) lifestyle modification, aimed to lose weight and physical activity, improved autonomic function indices (HR variation and QT indexes) more than metformin or placebo (91). A review published in 2007, has shown the improvement on autonomic function obtained from weight loss in individuals with diabetes and obesity (92). On the other hand, moderate endurance and aerobic exercise seem to improve cardiac autonomic function independent of BMI, blood pressure, glycemic control, and diabetes duration in patient with T1DM and T2DM as shown in a recent review in Ref. (93).

## Therapy

Once DAN becomes clinically evident, there is no treatment, which can effectively stop or reverse it. The most recent studies confirmed the efficacy of intensive insulin therapy in slowing the progression of both diabetic peripheral neuropathy (94) and DAN (95). This goal is obtained in T1DM by increasing the frequency of daily injections or by using a pump for continuous subcutaneous insulin infusion. In T2DM, several antihyperglycemic drugs, like sulfonylureas, GLP-1 agonists, thiazolidinediones, have shown beneficial effects in diabetes complications (51). In contrast, metformin seems to worsen neuropathic damage because of its effect on vitamin B12 (96). In particular, in the past years, attention has been paid to CAN outcomes. A number of treatments have been shown to target and contrast the pathogenetic pathways of CAN or to improve its symptoms. The efficacy of antioxidants like α-lipoic acid or vitamin E in increasing HRV is controversial (14). Aldose reductase inhibitors (ARIs) studies have shown disappointing results because of the poor effects and the induction of adverse events like hepatic and renal toxicity. Recent experimental studies on ARIs look promising but they need to be validated (51, 97). The use of agents inhibiting peroxynitrite formation (FP15 and FeTMPS) has recently been examined in diabetic rats with positive outcomes (8).

Furthermore, C-peptide has shown beneficial effects on HRV in T1DM patients as it enhanced endoneurial blood flow, Na^+^/K^+^ pump activity, and neurotrophic factors release (97). Similarly, HRV may be treated with antihypertensive drugs (ACE inhibitors, angiotensin receptor blockers, cardioselective β-blockers, digoxin, and verapamil) (8).

Symptomatic orthostatic hypotension therapy has been extensively investigated. When lifestyle, behavioral measures, and physical countermanoeuvres are no longer effective, pharmacological intervention should be considered. Although only midodrine, an α1-adrenergic agonist, has been approved by the Food and Drug Administration for the therapy of orthostatic hypotension, α-2 antagonists (clonidine), mineralocorticoids (9-α-fluorohydrocortisone), non-selective β-blockers, somatostatin and its analogs (octreotide), erythropoietin, desmopressin acetate, cholinesterase inhibitor (pyridostigmine bromide), caffeine, and acarbose have been found to ameliorate symptoms through different mechanisms, albeit with limited effectiveness (12, 14).

Inhibitors of specific antioxidant pathways, especially NF-kB and Nfr-2, mitochondria targeted antioxidants as well as enhancers of mitochondrial functions have been suggested as future strategies against DAN (50). Finally, new possibilities have been opened by stem cells and gene therapy (32).

## Conclusion

In conclusion, DAN is a particular aspect of diabetic neuropathy, which leads to multisystemic impairment in both T1DM and T2DM patients. Cardiac system is the most seriously involved.

The pathogenesis of DAN has yet to be clarified but metabolic, genetic, and hormonal factors have been reported. The final common effect seems to be hyperglycemia resulting in oxidative stress and inflammation.

As nowadays no therapy is able to effectively reverse this process, prevention with strict glycemic control, multifactorial intervention, and lifestyle modification remains essential.

## Conflict of Interest Statement

The authors declare that the research was conducted in the absence of any commercial or financial relationships that could be construed as a potential conflict of interest.
